# Small Molecule Protease Inhibitors as Model Peptidomimetics

**DOI:** 10.3390/ph18091377

**Published:** 2025-09-15

**Authors:** Patricia Gomez-Gutierrez, Juan J. Perez

**Affiliations:** Departament d’Enginyeria Química, Universitat Politècnica de Catalunya-Barcelona Tech, Edifici ETSEIB, Av. Diagonal 647, 08028 Barcelona, Catalonia, Spain; patricia.gomez-gutierrez@upc.edu

**Keywords:** protease inhibitors, isostere replacement, peptidomimetics, drug discovery, structure–activity relationships

## Abstract

Proteases constitute one of the largest sub-classes of enzymes, accounting for ca. 2% of the proteins encoded in the human genome. They play a key role in protein degradation and signaling, regulating a variety of physiological processes. Dysregulation of their activity is associated with various pathological conditions like cancer, neurodegenerative disorders, inflammatory or cardiovascular diseases. Protease activity can be controlled by regulating enzyme concentrations, but also by inhibitors, molecules that modulate enzyme function, inspiring the development of small molecule protease inhibitors for therapeutic purposes. Protease inhibitors can be designed from the corresponding substrates by isostere replacement at the scissile bond. This process yields a first-generation of inhibitors that usually exhibit poor drug-like profiles that need subsequently be improved to generate a second-generation, by smoothing their peptide-like features. This process is reviewed in the present report and exemplified in the successful discovery stories of different inhibitors that correspond to four types of proteases, including the angiotensin converting enzyme (metalloprotease); HIV protease (aspartate protease); thrombin (serine protease) and the proteasome (threonine protease). A detailed description of the stories behind their design from their initial discovery to the final product is described in this report. Moreover, despite successful discovery stories, the challenges associated with designing novel protease inhibitors are examined. Finally, the relevance of these drugs in the present drug market is also reported.

## 1. Introduction

Proteases, also called peptidases or proteinases, are hydrolases endowed to cleave a peptide bond from a peptide substrate. With about 2% of the proteins encoded in the human genome, proteases constitute one of the largest sub-classes of enzymes [1]. A key function of proteases is protein degradation, a process that is relevant to food digestion and intracellular protein turnover. In eukaryotes, this process is accomplished in specialized organelles called lysosomes by means of chaperone-mediated autophagy, although it can also be accomplished in the cytosol, mainly through the ubiquitin–proteasome system, in which proteins are selectively tagged with ubiquitin for degradation in the proteasome [2]. In addition, proteases can also act as signaling molecules by exercising their proteolytic activity on diverse substrates as posttranslational modifications, regulating a variety of physiological processes such as development, differentiation, cell migration, immunity, wound healing and cell death. Dysregulated proteolytic activity is associated with various pathological conditions like cancer, neurodegenerative disorders, inflammatory or cardiovascular diseases [3].

**Figure 1:**
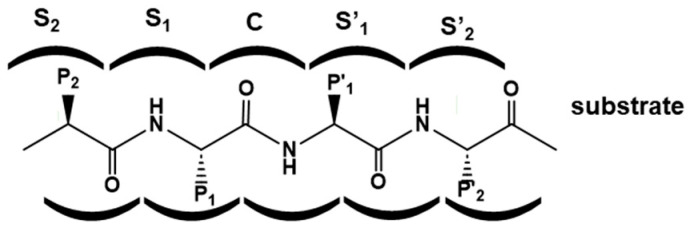
Enzyme binding site and sub-sites. Letter S represents enzyme sub-sites and letter P represents substrate side chains. Numeration regards the scissile bond sub-site C.

Substrates bind to the protease binding site—a groove that accommodates the peptide chain—in a way that the hydrolysis of the scissile bond can be accomplished. The binding site can be described as a cavity with several subsites that accommodates a single substrate residue side chain. These subsites are labeled according to their distance to the scissile bond as S_1_–S_n_ in ascending order towards the N-terminus of the substrate and as S_1_′–S_n_′ in ascending order towards the C-terminus, taking the scissile bond as reference (Figure 1). Conversely, the corresponding substrate residues accommodated in the different subsites of the groove are numbered following the same scheme as P_1_–P_n_, and P_1_′–P_n_′, respectively [4].

**Figure 2:**
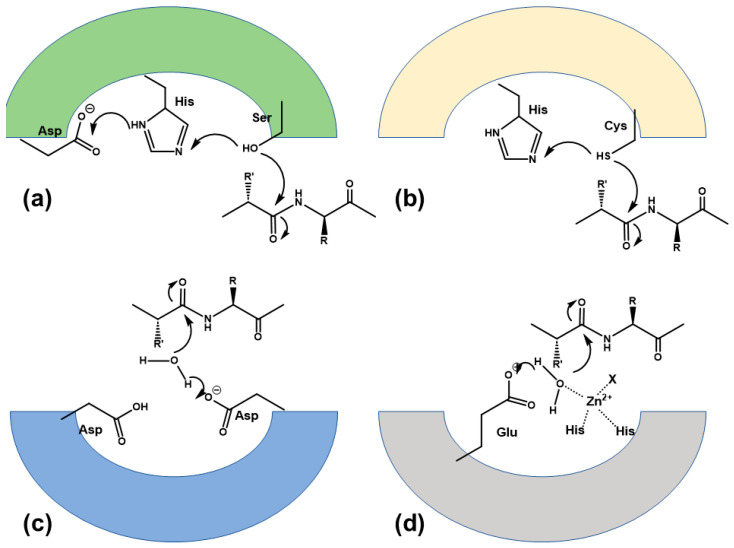
Cleavage of the peptide bond by different types of proteases. (**a**) serine protease; (**b**) cysteine protease; (**c**) aspartic protease; (**d**) metalloprotease.

Cleavage of the peptide bond takes place through a nucleophilic attack on the carbonyl amide, either by an activated alcohol or thiol of a sidechain or directly, by an activated water molecule. The differential mechanism permits to classify proteases into two broad subtypes. In the first subtype, the mechanism proceeds through the nucleophilic attack to the carbonyl amide by an activated oxygen of a serine (serine proteases), a threonine (threonine proteases) or the sulfur of a cysteine (cysteine proteases) with the formation of a transient covalent bond between the substrate and the enzyme nucleophile (Figure 2a,b). In the second subtype, an activated oxygen from a water molecule acts as a nucleophile. In this case, no transient covalent bond with the protease is formed but a geminal diol-type intermediary is formed. The mechanism may be assisted by a metal cation, as in metalloproteases, or proceed without one, as in aspartic and glutamic proteases (Figure 2c,d).

Tight regulation of proteases is essential for maintaining cell homeostasis, since imbalances can lead to pathological conditions. Regulation of protease concentrations can be achieved through gene expression or trafficking but also by limiting its maturation, since most proteases are expressed as inactive zymogens or through the inhibition of posttranslational modifications. In addition, pre-existing enzyme activity can be modulated by means of inhibitors [5]. Endogenous inhibitors circulate in plasma and inactivate hemostatic system proteases after their proteolytic activity is largely complete. Thus, enzyme inhibition serves as an effective regulatory mechanism in cells that can be leveraged for therapeutic intervention [6,7,8]. Presently, about 10% of marketed drugs are small molecule protease inhibitors, covering diverse therapeutic areas that range from antiviral agents to regulators of blood clot formation or blood pressure, as well as anticancer agents.

Despite the demonstrated success, the design of protease inhibitors is not without significant challenges. Successful protease inhibitors are expected to be selective, since non-specific inhibitors can affect off-target enzymes leading to side effects. In addition, a thorough understanding of the target enzyme biology is required to avoid undesirable side effects. Essentially, poor knowledge of the biology together with the design of non-selective inhibitors are the reasons behind the abandon of several discovery programs on matrix metalloproteases (MMPs) inhibitors for cancer treatment in the mid-90s [8]. Although a series of inhibitors were discovered, they failed in clinical trials due to lack of selectivity and to the overlapping mechanisms of diverse MMPs in matrix turnover. Another challenge already mentioned in this paper is the improvement of a poor pharmacokinetics profile and its oral bioavailability. This can be achieved by smoothing the peptide-like features that inhibitors may exhibit. Finally, specific inhibitors may develop resistance due to the emergence mutant proteases that decrease their affinity, lowering their efficacy. This can be exemplified in the process behind the development of HIV protease inhibitors due to the huge capacity of the virus to mutate its genes. To overcome this challenge, a design strategy based on using protein backbone anchoring points rather than residue side chains permitted to yield satisfactory compounds.

The present review examines the process of designing selective protease inhibitors considered within the framework of peptidomimetics derived from their native peptide substrates. As described below, first-generation small molecule inhibitors can be generated by replacement of the scissile peptide bond of a minimal length substrate by diverse isosteres. Then, structure–activity studies are eventually carried out to increase their potency and selectivity. Subsequently, a second generation of inhibitors is normally produced in order to improve their ADME profile by eliminating peptide features they may exhibit. The design process is exemplified herein through the account of the research pathway that culminated in the discovery of different protease inhibitors of diverse targets, including the angiotensin converting enzyme for the treatment of congestive heart failure and high blood pressure; the HIV protease for the treatment of AIDS; thrombin to manage blood clot formation or the proteasome to treat multiple myeloma.

## 2. Designing Protease Inhibitors

Features of a protease active subsite determine the binding residues complementary to the substrate and consequently, determine its specificity. Accordingly, characterization of these subsites is of paramount importance for designing selective inhibitors since they differ even in proteases of the same family [9]. The 3D structures of a number of protease-inhibitor complexes solved by X-ray diffraction or NMR spectroscopy are compiled, among other peptide–protein complexes in the PepBind data base [10]. Analysis of the structures suggests that protease cleavage sites are frequently located in flexible solvent-exposed loops, although a number of sites have also been identified in α–helix structures. In general, enzyme binding sites are flexible regions, explaining why only a few structures that include residues encompassing the cleavage site have been solved by X-ray crystallography [11]. Inhibitors most frequently bind in extended conformations like in the case of the HIV protease [12], although bent structures can also be found like the urokinase plasminogen activator inhibitor [13]. Moreover, some peptides exhibit a helical structure like the calpain inhibitors [14] and even hairpin structures like in the β-trypsin inhibitor [15].

**Figure 3:**
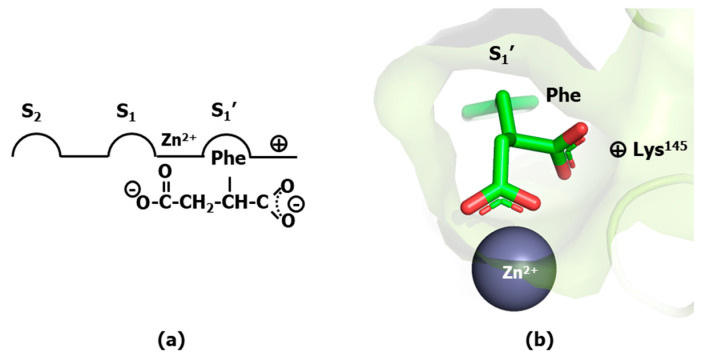
Binding mode of L-benzylsuccinate to carboxypeptidase A. (**a**) schematic representation of ligand occupation of the diverse enzyme subsites. (**b**) 3D display of the ligand binding mode using the crystallographic structure (pdb entry 1CBX).

Aside from the direct procedure of hit identification and growth at the active site of a specific protease [16], a systematic successful strategy to design small molecule protease inhibitors starts from the shortest possible peptide substrate and modify its structure by replacing the scissile peptide bond by a non-liable isostere to hamper its hydrolysis. Moreover, isosteres embedding features of the transition state are expected to exhibit larger affinities than substrates [17]. In the case of protease inhibitors, since their bioactive conformation is most frequently extended, the process of peptide bond isostere replacement is demonstrated to be a good strategy for designing first-generation peptidomimetics, although not necessarily with higher affinities than the original peptide [18]. The first successful application of this strategy was the identification of L-benzylsuccinate as a potent inhibitor of carboxypeptidase A [19], a metalloprotease that uses Zn^2+^ to hydrolyze peptide bonds of C-terminal residues with aromatic or aliphatic sidechains. The inhibitory capacity of L-benzylsuccinate can be explained assuming that the ligand binds to the enzyme active site with one of carboxyl groups acting as phenylalanine C-terminus occupying the S_1_′ subsite, whereas the other carboxyl group chelates the metal ion (Figure 3a). L-benzylsuccinate binds 160 times more tightly than 3-phenylpropionate and 3000 times more tightly than the substrate carbobenzyloxyglycylglycyl-L-phenylalanine, underlying the importance of the second carboxyl group of the molecule. The hypothesis was later confirmed by X-ray crystallography (Figure 3b) [20]. Specifically, analysis of the structure reveals the inhibitor with one of carboxyl groups acting as phenylalanine C-terminus, accommodated in the S_1_′ subsite defined by the guanidinium group of Arg^145^ and the side chains of Tyr^248^ and Asn^144^ and with the other carboxyl group forming a bidentate bond to the metal ion, mimicking the concomitant interactions of the carbonyl substrate and oxygen water molecule in the transition-state. The use of a chelating moiety to mimic the transition state result was subsequently used as common strategy for metalloprotease inhibitor design [21].

## 3. Peptide Bond Isostere Replacement

**Figure 4:**
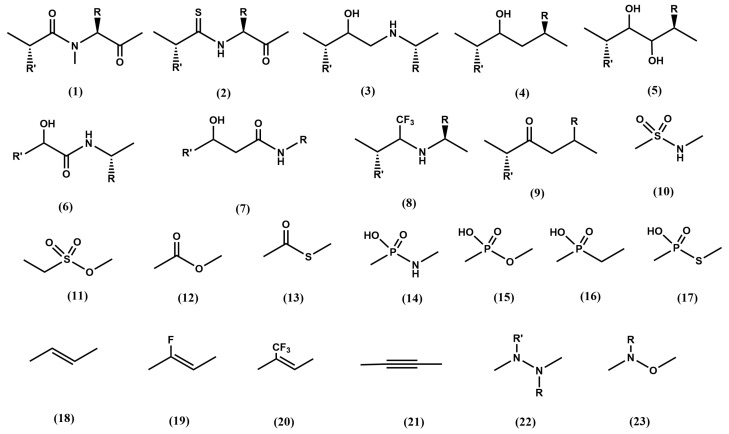
Chemical structures of diverse peptide bond isosteres in reference to the standard peptide bond (see text).

Diverse peptide bond isosteres have been reported in the literature for designing protease inhibitors [22]. Figure 4 illustrates common peptide bond isosteres, which can be regarded as modifications to prevent peptide bond liability. A conservative modification of the peptide bond is exemplified by the N-methylamides (**1**) [23]. N-methylamino acids are found in diverse non-ribosomal peptides in nature, like cyclosporine A. N-methylation is a substitution that stabilizes the peptide against enzymatic degradation, improves its pharmacokinetic profile and also helps to improve its bioavailability. From the structural point of view, N-methylation introduces important changes on the conformational profile of the peptide since the loss of the hydrogen bond capability of the amide group may disrupt any secondary structure motif involving the group. Moreover, methylation of the amide group facilitates the occurrence of a *cis* bond on the preceding peptide bond and increases the basicity of the carbonyl group. Another conservative modification of the peptide bond involves replacing the carbonyl oxygen with sulfur, resulting in a thioamide (**2**). Differences with the oxoamide are subtle, including being a weaker hydrogen bond acceptor, although a stronger hydrogen donor or exhibiting a higher peptide bond rotational barrier [24]. The thioamide moiety is also found in natural peptides like thioviridamide, a bacterial apoptosis inducer [25].

The substitution of the carbonyl moiety of the scissile peptide bond by an alcohol to mimic the tetrahedral intermediate of the hydrolysis process is a judicious choice for designing transition state analogs. However, reduction of the amide carbonyl to alcohol produces a non-stable hemiaminal derivative that is easily transformed into an imine after a water molecule loss. Instead, a closer stable isostere containing the alcohol function can be made by introducing an extra methylene group between the alcohol moiety and the amide nitrogen to generate hydroxyethylamine (**3**) derivatives. This isostere introduces higher conformational flexibility to the original peptide bond due to the loss of its partial double bond character, helping the inhibitor to better accommodate to the features of the protease active site groove. This motif has successfully been used in the development of potent aspartic protease inhibitors, like the HIV protease for the treatment of AIDS [26], β-secretase for the treatment of Alzheimer disease [27,28] or plasmepsin X for the treatment of malaria [29].

An alternative isostere can be produced by reduction of the peptide bond carbonyl to alcohol with a simultaneous substitution of the amide nitrogen by a methylene to give the hydroxyethylene (**4**) and dihydroxyethylene (**5**) isosteres. These isosteres do not introduce an extra methylene in the chain, keeping the conformational freedom of the hydroxyethylamine derivatives. Examples where this isostere was incorporated are aliskiren, a potent inhibitor of renin (an aspartic protease) for the treatment of hypertension [30] or more recently, in the design of inhibitors of metallopeptidase dipeptidyl peptidase III for the improvement of cardiac and renal function after a heart failure [31].

Similarly, interesting isosteres can be constructed by incorporating an alcohol function to β or γ amino acids. Thus, the hydroxymethylcarbonyl isostere (**6**) exhibits the alcohol function in position α of a β-amino acid and in the hydroxyethylcarbonyl isostere (**7**) the alcohol function is located in position β of a γ amino acid. Both isosteres receive inspiration from the unnatural amino acid statine (3S,4S)-4-amino-3-hydroxy-6-methylheptanoic acid), found in the natural aspartic protease inhibitor pepstatin, isolated from cultures of various species of actinomyces. The hydroxymethylcarbonyl isostere was incorporated in the design of KRI-1314, a potent renin inhibitor for the treatment of hypertension [32], as well as in the design of inhibitors of the HIV-1 protease, β-secretase, and HTLV-I protease [33]. Moreover, the two isosteres were used in the design of plasmepsins [34].

Alternatively, the amide carbonyl can be replaced by a trifluoro moiety, giving rise to the trifluoroethylamine isosteres (**8**). This represents an interesting substitution since the trifluoromethyl moiety is isopolar to the carbonyl group. Another feature of the isostere is the decreased basicity of the amine without compromising the ability of the NH to function as a hydrogen bond donor. The trifluoroethylamine isostere was employed in the design of odanacatib, a cathepsin K inhibitor used to prevent calcium resorption for the treatment of osteoporosis [35]. Despite its demonstrated effectivity, its development was discontinued after phase III clinical trials due to a demonstrated risk increase in stroke.

Replacement of the peptide bond nitrogen by a carbon produces the ketomethylene isostere (**9**), lifting the *cis*/*trans* constraint of the peptide bond. Inhibitors with this isostere act as substrates and then, subject to a nucleophilic attack on the ketomethylene carbonyl produce a stable tetrahedral intermediate. This can be useful for those enzymes that use a water molecule as nucleophile. Specifically, this isostere has been successfully used for designing aspartic protease inhibitors, like in the case of the HIV protease [36] or calpain inhibitors [37]. Moreover, the methylene group can be further substituted by fluorine atoms that activate the electrophilic carbonyl group and exhibit higher reactivity, giving rise to α-fluorinated ketone isosteres. Analogs containing this isostere have also been used to design HIV protease inhibitors [38]. In a step further, the α,α-difluoro-β-hydroxyketone unit [39] was also explored in the design of protease inhibitors, such as HIV-1 protease [40], elastase [41], and human heart chymase [42].

The carbon atom of the carbonyl group can be replaced by similar chemical groups using other heteroatoms, like sulfur or phosphorus. Interestingly, these isosteres exhibit a tetrahedral configuration suitable to act as transition state analogs. In the case of sulfur, isosteres like the sulfonamide and sulfonate can be produced. Sulfonamides (**10**) have extensively been used as inhibitors of matrix metalloproteases, enzymes playing a central role in extracellular matrix turnover with interesting antitumor properties [43]. The isostere has also been used to design amprenavir, a HIV protease inhibitor [44]. Sulfonates (**11**) have been used as inhibitors of cysteine proteases [45]. Moreover, substitution of the amine nitrogen by oxygen or sulfur produces derivatives like the methyleneoxy isostere (**12**) or the methylenemercapto isostere (**13**), respectively. These are less attractive isosteres due to the loss of the hydrogen bond accepting capability and they are more easily hydrolyzed than their amide counterparts [24]. Substitution of the carbonyl carbon by phosphorus produces isosteres with similar characteristics to the sulfur containing isosteres described above, like the phosphonamide (**14**), phosphonate (**15**), phosphinate (**16**) or phosphothionate (**17**) [46,47].

Although alkanes can also be considered isosteres of the peptide bond [48], more interesting is when they are reduced to alkenes (**18**) [49] as well as their flourine derivatives—the fluoroalkene (**19**) and the trifluoroalkene (**20**) isosteres [50]. Despite preserving the planarity of the peptide bond with the *cis*/*trans* isomerism associated, they exhibit smaller dipole moments and loss of the ability to participate in hydrogen bonding. These differences have consequences in the structural features of the sequences they participate. For example, they are expected to be helical disruptors. Further reduction of the peptide bond produces alkynes (**21**) that have also been used as peptide bond isosteres [51]. Other isosteres that recover the amide group at the expense of losing the alpha carbon are the hydrazine (**22**) [52] and the amideoxy isostere (**23**) [53].

## 4. Protease Inhibitor Design Case Studies

In this section, we describe the stories behind the development of potent inhibitors of different types of proteases including a metalloprotease (the angiotensin converting enzyme), an aspartyl protease (the HIV protease), a serine protease (thrombin) and the proteasome, a molecular complex with multiple threonine protease sites. These case studies constitute paradigmatic examples of protease inhibitor discovery, illustrating the use of general strategies on peptidomimetics design to reach small molecule protease inhibitors.

### 4.1. Angiotensin-1 Converting Enzyme Inhibitors

The design of angiotensin-1 converting enzyme (ACE) inhibitors is an early example of successful metalloprotease inhibitor design, with the first compounds disclosed at the beginning of the 1970s. It should be stressed that many of the tools available today were not mature enough at that time, so a deep recognition needs to be paid to this pioneering work in drug discovery [54].

Hypertension is a chronic medical condition in which the blood pressure in the arteries is elevated. It is considered the most common disease affecting the heart and blood vessels, afflicting about a fourth of the adult population throughout the world [55]. Hypertension can damage organs and be the origin of diverse illnesses such as, renal failure, aneurysm, heart failure, stroke, or heart attack. Antihypertensive drugs are therapeutic agents used to lower blood pressure. The types of antihypertensives currently marketed act through different mechanisms of action including diuretics, which decrease blood volume by promoting elimination of water in the urine; modulators of the renin–angiotensin system like β-blockers; ACE inhibitors or angiotensin II antagonists; and calcium channel blockers that slow the influx of calcium into vascular smooth muscle fibers [56].

Angiotensin II is a potent vasoconstrictor and the final product of the renin–angiotensin system designed to control water and electrolyte homeostasis, stimulating the secretion of aldosterone from the adrenal cortex by increasing sodium reabsorption in kidneys [57]. ACE is a zinc metalloprotease that processes angiotensin I into angiotensin II by cleavage of the two C-terminal residues. Inhibition of the enzyme prevents angiotensin II release and consequently avoids the increase in blood pressure levels. Presently, there are seventeen non-peptide ACE inhibitors commercially available. They can be classified into three categories, depending on the way they bind to the zinc ion, including mercaptans, carboxyalkyl dipeptides and phosphinic acids [58,59].

The discovery of the first ACE inhibitors is associated with the characterization of the Brazilian pit viper *Bothrops jararaca* venom at the beginning of the 1970s. At that time, it was known that the venom contains a component that greatly enhances the smooth-muscle-relaxing action of bradykinin. Diverse studies showed later that the pharmacological action of the venom is mediated through inhibition of the ACE protease that prevents the release of angiotensin II, avoiding its potent vasoconstrictor action and simultaneously bradykinin degradation enhancing its muscle relaxant action. Analysis of the venom components revealed different peptides with potent ACE inhibition capacity. Among them, the pentapeptide pGlu-Lys-Trp-Ala-Pro was found to have the highest affinity for the enzyme and the nonapeptide teprotide, with sequence pGlu-Trp-Pro-Arg-Pro-Gln-Ile-Pro-Pro found to have the highest ACE-inhibitory activity duration in vivo [60]. The latter sequence was subsequently chosen as a proof-of-principle, to demonstrate the blood-pressure-lowering activity of these peptides in hypertensive patients [61]. Although the results were encouraging, its poor oral bioavailability precluded its use as therapeutic agent. An orally bioavailable drug was subsequently pursued by designing small molecule peptidomimetics of these peptides.

**Figure 5:**
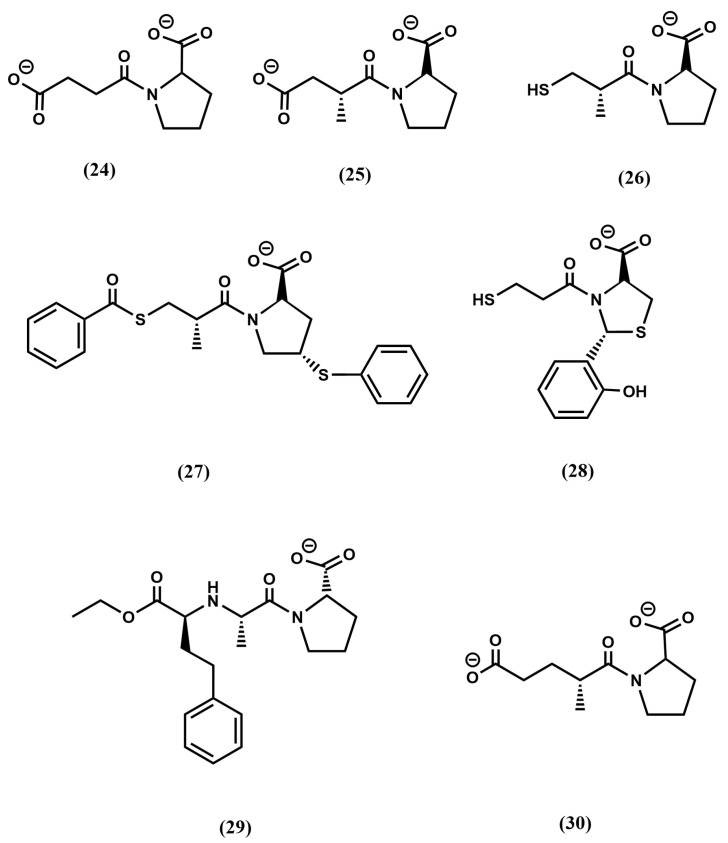
Chemical structures of diverse ACE inhibitors (see text).

**Figure 6:**
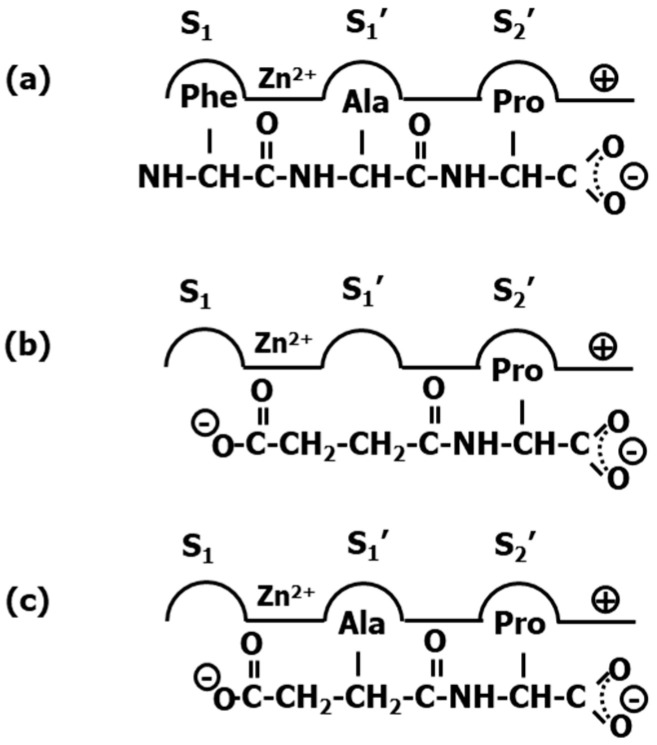
Schematic representation of ligand occupation of the diverse enzyme subsites. (**a**) tripeptide Phe-Ala-Pro; (**b**) succinyl-proline dipeptide (**24**); (**c**) D-2-methylsuccinyl-L-proline (**25**).

Without any knowledge about the 3D structure of the ACE-teprotide complex, it was imperative to follow an indirect design approach. Accordingly, a necessary first step to acquire an understanding of the features of the ligand–receptor interaction came from structure–activity studies on teprotide analogs. These studies permitted to identify the tripeptide Phe-Ala-Pro as the minimal active C-terminal fragment that was used subsequently as reference model. Moreover, since ACE is a zinc metalloprotease, inspired on the success of the discovery of L-benzylsuccinic acid as a potent inhibitor of carboxypeptidase A [19], the succinyl-proline dipeptide (**24**) and its α-methyl derivative, the D-2-methylsuccinyl-L-proline (**25**) were used as mimetics of the last two residues of the reference model tripeptide and subsequently synthesized and tested [62] (Figure 5). Although both compounds exhibited less potency than teprotide, they were shown to be specific binders in the smooth muscle contraction assays. The partial success of compound **25** permitted to work out a hypothesis of the binding of these derivatives to the enzyme active site. First, it was hypothesized that the peptide Phe-Ala-Pro binds to the enzyme active site so that the proline side chain is accommodated in the subsite S2′, the Ala side chain in the subsite S1′ and the phenylalanine side chain in the subsite S1, as shown in Figure 6a. Moreover, derivatives **24** and **25** were assumed to bind in such a way that the carbonyl of the succinic acid at the N-terminus interacts with the zinc ion of the metalloprotease (Figure 6b,c). Subsequent substitutions on **25** aimed at improving its chelating capacity, led to the discovery of the sulfhydryl analog D-3-mercapto-2-methylpropanoyl-proline (captopril) (**26**) (Figure 5) with a 1000-fold increase in inhibitory potency. The resulting compound proved to be one of the first antihypertensive small molecule designed to bind to the active site of ACE [62]. Following a medicinal chemistry approach, diverse captopril analogs were subsequently synthesized, providing useful information about the pharmacophoric features of the binding site. These studies led to the design of zofenopril (**27**) [63] and rentiapril (**28**) [64], approved a few years later (Figure 5). These three compounds all have the thiolate group as a zinc ion binder in common.

**Figure 7:**
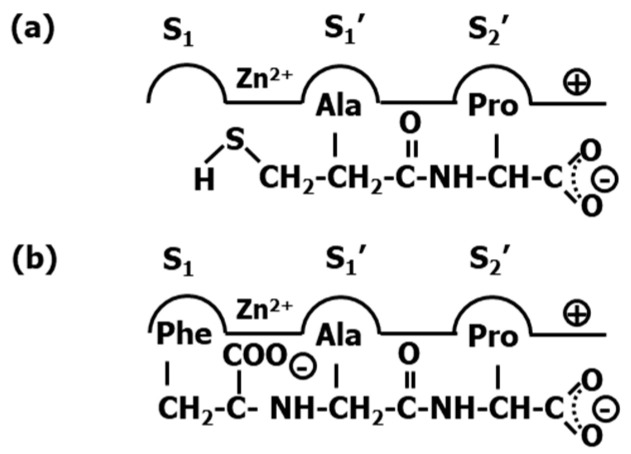
Schematic representation of ligand occupation of the diverse enzyme subsites. (**a**) captopril (**26**); (**b**) enalapril (**29**).

Captopril and its analogs exhibit adverse side effects, such as skin rash and some taste disturbances, putatively caused by the mercapto-containing penicillamine. This prompted to search for novel ACE inhibitors through reversion to use a carboxyl moiety as part of the chelating group (Figure 7a). Extensive medicinal chemistry studies led to the discovery of enalapril (**29**) (Figure 5), the first member of a series of carboxyalkyl-dipeptides with a high ACE inhibitory profile [65]. The discovery of enalapril started by the identification of compound D-2-methylglutaryl-L-proline (**30**) (Figure 5), using the Pro C-terminal residue of the model tripeptide as reference, as worked out in compound **26**. Modification of the methylene in position 3 by a secondary amine resulted in a compound that was significantly improved further when a methyl group was added in position 4. Finally, inspired by the model tripeptide, it was found that the phenylethyl group was optimal for a good inhibitory capacity (Figure 7b).

**Figure 8:**
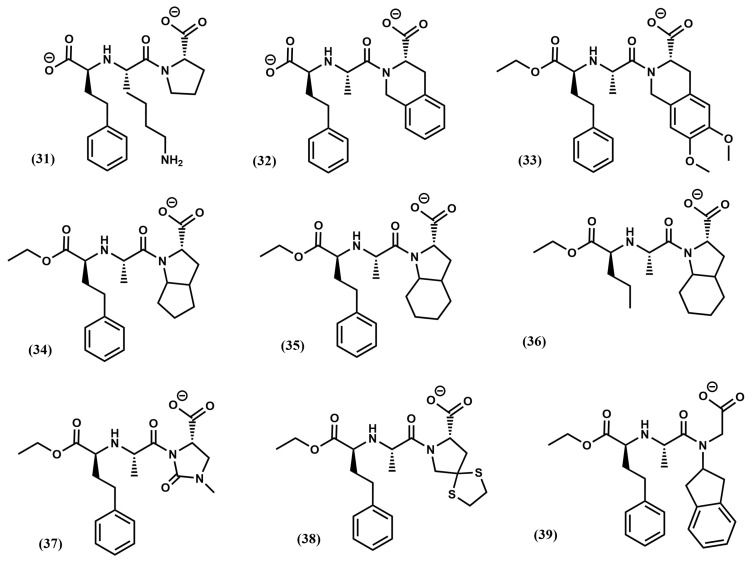
Chemical structures of diverse ACE inhibitors (cont.) (see text).

**Figure 9:**
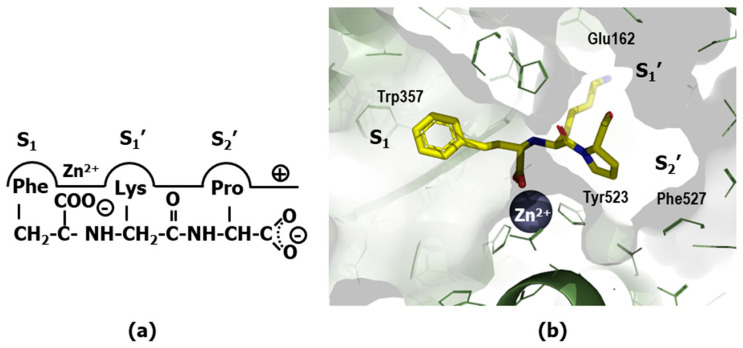
Binding mode of lisinopril to ACE. (**a**) schematic representation of ligand occupation of the diverse enzyme subsites. (**b**) 3D display of the ligand binding mode using the crystallographic structure (pdb entry 1O86).

After the success of enalapril, several compounds were subsequently designed. Notably, the substitution of the alanine residue by a lysine produced lisinopril (**31**) (Figure 8) that resulted in a maximal accommodation of the inhibitor to the ACE active site and in improvement of the oral properties of the drug [66]. At the beginning of the 2000s, the crystallographic structure of lisinopril bound to the protease was the first ACE-inhibitor structure released [67]. Analysis of the structure confirms that the inhibitor makes extensive contacts with the active site residues according to the hypothesis generated to explain binding of captopril (Figure 9a). Specifically, the carboxyalkyl carboxylate of the ligand chelates the zinc ion located on active site of the metalloprotease as expected, together with three other coordination points including His^383^, His^387^ and Glu^411^. Moreover, the phenylethyl group occupies the S_1_ pocket defined by residues Trp^357^, Phe^512^ and Val^518^, the lysynyl amine appears accommodated in the S_1_′ pocket, forming a weak H-bond with Glu^162^ and the proline sidechain occupies the S_2_′ pocket defined by a bunch of aromatic residues, including Phe^457^, Tyr^520^, Tyr^523^ and Phe^527^. Finally, the C-terminal carboxylate exhibits interactions with Lys^511^ and Tyr^520^ (Figure 9b).

**Figure 10:**
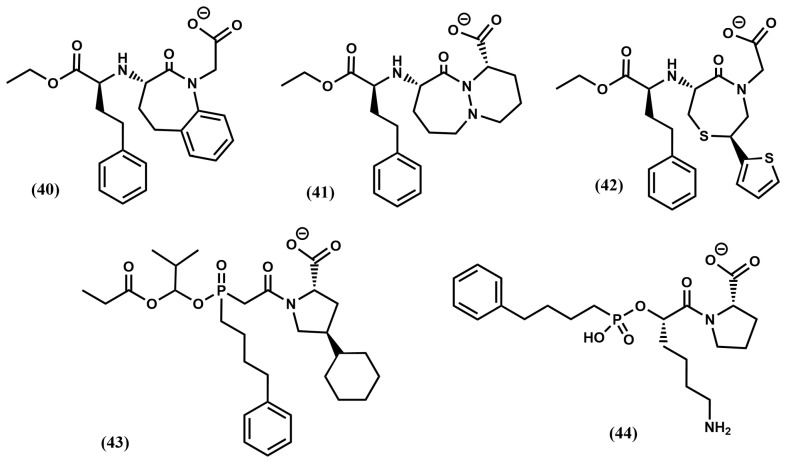
Chemical structures of diverse ACE inhibitors (cont.) (see text).

Diverse analogs of enalapril were subsequently designed (see Figure 8), some of them consisting of diverse ring substitutions of the P_2_′ moiety, such as quinapril (**32**), moexipril (**33**); ramipril (**34**), trandolapril (**35**) and perindopril (**36**), its analog with the phenyl ring removed, imidapril (**37**), spirapril (**38**) or delapril (**39**). Conformationally constrained analogs were also synthesized including benazepril (**40**), cilazapril (**41**) or timocapril (**42**) (Figure 10). Finally, a third group of ACE inhibitors was designed using a phosphinate group to chelate the zinc ion of the metalloprotease. Compounds in this class include fosinopril (**43**) [68] and ceronapril (**44**) [69] (Figure 10).

The ACE inhibitors commercially available discussed above, are referred in the literature as first-generation ACE inhibitors, since they all were developed prior to the current knowledge on the enzyme. ACE was sequenced at the beginning of the 1990s [70] and subsequently, cloned and expressed [71], and the first crystallographic structure of the enzyme was released at the beginning of the 2000s [67]. Intriguingly, somatic ACE exhibits two catalytically active large homologous domains referred as N- and C-domains that exhibit different functions in vivo, with the former engaged in the regulation of hematopoietic stem cell proliferation and the latter, involved primarily in the control of blood pressure [59]. This differential functionality is the result of a distinctive substrate specificity between domains that interact differently with competitive inhibitors [72], modulating the levels of non-angiotensin I substrates like bradykinin or the Ac-Ser-Asp-Lys-Pro peptide. This discovery offered an opportunity to design second-generation ACE inhibitors with specific profiles [73,74]. However, in spite that a few domain-selective ACE inhibitors are currently available, their poor pharmacokinetic profile precludes fully understanding of their clinical significance [75].

### 4.2. HIV-1 Protease Inhibitors

Acquired immunodeficiency syndrome (AIDS) is an illness caused by the human immunodeficiency virus (HIV-1). The virus infects CD4+ lymphocytes and causes their destruction with the subsequent weakening of the immune system. In these conditions, the body becomes progressively susceptible to opportunistic infections. Since the identification of the HIV-1 virus at the beginning of the 1980s as responsible for the infection, the number of people infected with the virus has grown steadily, with a present annual rate of 1.3 million people. In 2024, the WHO estimated about 40.8 million people infected worldwide and 650,000 people accounted died from AIDS-related illnesses [76]. A large number of biological and biochemical studies conducted in recent years have shed light about the host cell entry mechanism and life-cycle of the virus, providing opportunities for controlling and eradicating the AIDS epidemics. Thus, drug candidates targeting diverse critical steps of virus maturation have been designed including entry inhibitors, nucleoside and non-nucleoside reverse-transcriptase inhibitors, integrase inhibitors, ribonuclease inhibitors and protease inhibitors [77]. However, due to the easy mutation of the virus into drug resistant forms, multiple drugs acting on different viral targets are used in a combined therapeutic action, known as highly active antiretroviral therapy (HAART). As a result of these efforts, AIDS has changed from being an acute and potentially life-threatening illness to a manageable chronic disease [78]. In this section the studies that led to the discovery of potent HIV protease inhibitors will be discussed. This represents a paradigmatic example of rational peptidomimetic design.

The HIV-1 aspartic protease is responsible for processing the viral polypeptide chain necessary for the organization of structural proteins and the release of viral enzymes during infection. Indeed, inhibition of the HIV protease prevents the conversion of the polyprotein genes gag and gag-pol into structural and functional entities, leading to the formation of immature and non-infectious virus particles.

**Figure 11:**
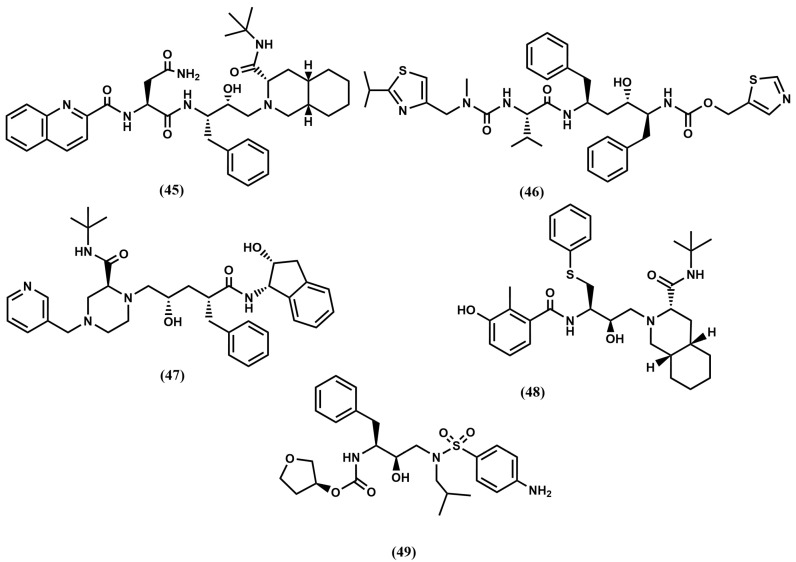
Chemical structures of diverse HIV protease inhibitors (see text).

The first HIV-1 protease inhibitors were designed by replacement of the substrate scissile bond by peptide bond isosteres. Beginning with the information that the HIV-1 protease cleaves Tyr-Pro/Phe-Pro sequences of the viral polypeptide, diverse peptide analogs containing a reduced amide or the hydroxyethylamine isostere were synthesized and tested. These studies led to the discovery of the first compounds with inhibition constants in the nanomolar range [79]. These compounds are peptide-like molecules that demonstrated proof-of-concept, but required further development to optimize their pharmacokinetic profile through the elimination of specific peptide characteristics. Refinement was carried out using a combined approach involving modeling, medicinal chemistry and X-ray crystallography [80]. A key information came with the release of the apo-protease 3D structure [81] and immediately after, a few structures of the enzyme/inhibitor complex including substrate analogs with the scissile bond replaced with a peptide bond isostere including the Ace-Thr-Ile-Nle-*ψ*[CH2-NH]-Nle-Gln-ArgNH2 (MVT-101) [82] and the Ac-Ser-Leu-Asn-Phe-*ψ* [CH(OH)CH2N]-Pro-Ile-Val-OMe (JG-365) [83]. Analysis of the structures provided valuable information to speed up the inhibitor design process. Specifically, the 3D structure of HIV protease is a C2-symmetrical homodimer composed of two identical 99-amino-acid chains. Its active site is located at the interface of the two subunits, each contributing a catalytic Asp^25^ residue, which are crucial for initiating protease activity. Interestingly, the structure reveals that each monomer exhibits a flap—a glycine rich region—which conformation changes significantly when the inhibitor is bound. The binding cleft contains hydrophobic residues and is large enough to accommodate peptides of approximately six to eight amino acids long. Moreover, features of the different enzyme binding subsites were identified and used to guide the design of new analogs within a specific architecture. The product of this research, the first generation of HIV-1 protease inhibitors was disclosed and subsequently approved for the treatment of AIDS. These compounds are based on hydroxyethylamine (**3**) and hydroxyethylene (**4**) isosteres with the central hydroxyl group mimicking the transition state. Compounds in this group are shown in Figure 11, including saquinavir (**45**), ritonavir (**46**), indinavir (**47**), nelfinavir (**48**) and amprenavir (**49**), as well as its phosphate ester, fosamprenavir, approved as a different drug. Despite the clinical success, further improvement was necessary due to the poor pharmacokinetic profile and associated toxicity exhibited by these inhibitors [84]. Moreover, their clinical use revealed the emergence of multidrug-resistant strains.

**Figure 12:**
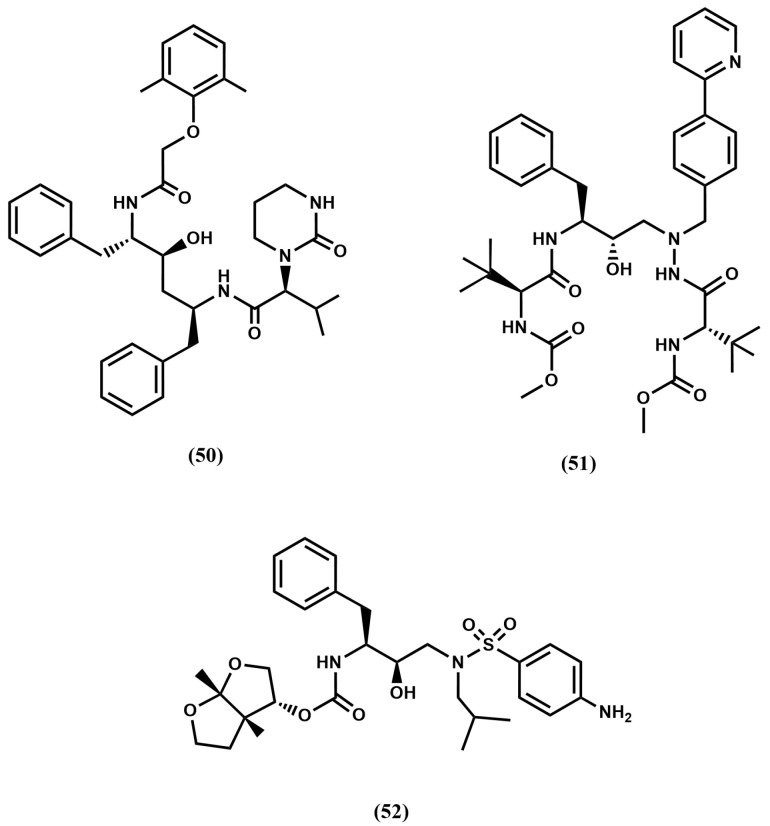
Chemical structures of diverse HIV protease inhibitors (cont.) (see text).

**Figure 13:**
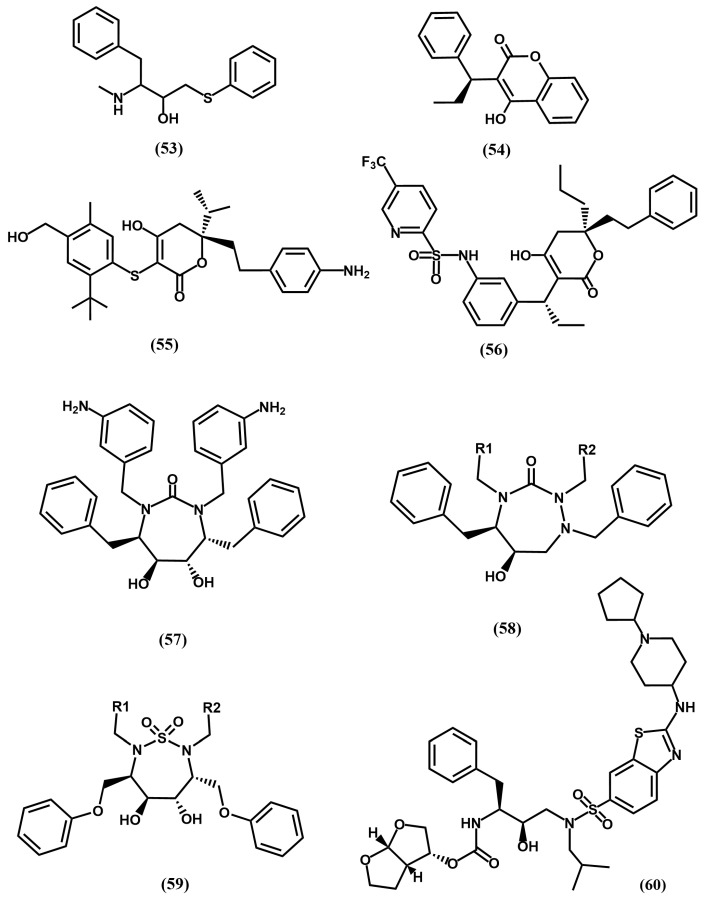
Chemical structures of diverse HIV protease inhibitors (cont.) (see text).

In order to avoid multidrug-resistant strains, a second generation of HIV protease inhibitors was developed using as design strategy favoring ligand–enzyme backbone interactions, considering that these interactions will remain unaffected by mutations. Following this strategy several commercially available protease inhibitors were subsequently disclosed including lopinavir (**50**), derived from ritonavir (**46**) and atazanavir (**51**) or darunavir (**52**), derived from amprenavir (**49**) (See Figure 12). Concurrently, the discovery of two hits—weak inhibitors of HIV-1 protease from separate screening programs—strengthened the search for novel second-generation inhibitors. On the one hand, the 4-hydroxy-6-phenyl-3-(phenylthio)-pyran-2-one (**53**), identified from a screening program at Parke-Davis and recognized as a conformationally constrained P_1_–P_1_′ dipeptide peptidomimetic [85]. On the other, the long-acting oral anticoagulant phenprocoumon (**54**) in a screening effort at Upjohn [86] (see Figure 13).

**Figure 14:**
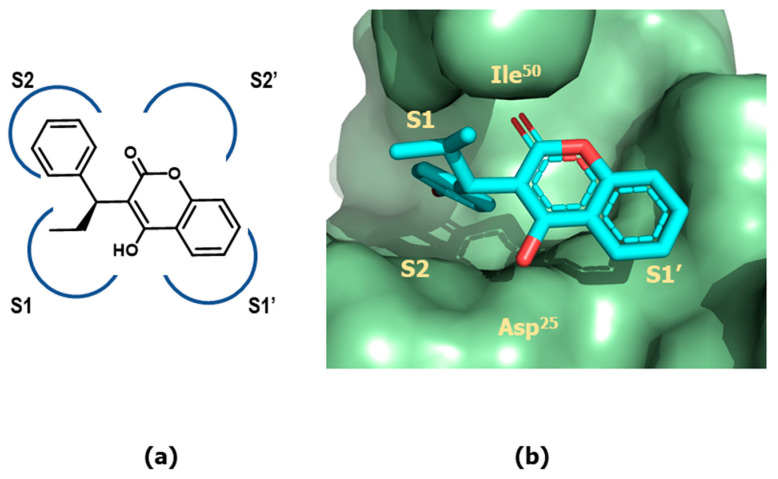
Binding mode of phenprocoumon (**54**) to the HIV protease. (**a**) schematic representation of ligand occupation of the diverse enzyme subsites. (**b**) 3D display of the ligand binding mode using the crystallographic structure (pdb entry 1UPJ).

The crystallographic structure of the latter bound to the HIV protease revealed the 4-hydroxy group forming hydrogen-bonds with the two catalytic Asp^25^/Asp^25^′ at the cleavage site and the lactone moiety interacting directly with the flap region residues Ile^50^/Ile^50^′, replacing a structural water molecule that mediated contacts between the carbonyl oxygen atoms and the amide group of Ile^50^ of the transition-state analog inhibitors described in the previous section. Furthermore, the α-ethyl and α-phenyl groups are accommodated on the S_1_ and S_2_ subsites. Moreover, the fused phenyl ring of the 4-hydroxycoumarin scaffold is placed in such a way that prevents the extension of the molecule to reach the S_2_′ subsite (Figure 14). It was then considered that removal of the fused ring to yield a 4-hydroxy-2-pyrone ring could offer many more possibilities for molecule extension. Interestingly, both lines of investigation converged to design a lead based on the 4-hydroxy-pyranone scaffold. Studies carried out at Parke-Davis led to interesting compounds like CI1029 (**55**) [87,88], whereas studies carried out at Upjohn resulted in tipranavir (**56**) [89] (Figure 13).

**Figure 15:**
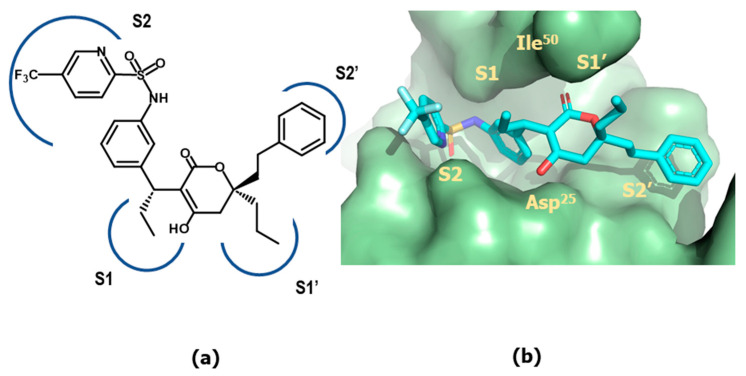
Binding mode of tipranavir (**56**) to the HIV protease. (**a**) schematic representation of ligand occupation of the diverse enzyme subsites. (**b**) 3D display of the ligand binding mode using the crystallographic structure (pdb entry 2O4P).

The X-ray crystallographic structure of the tipranavir/protease complex reveals that the ligand binds like the previously studied 4-hydroxypyranones [90]. The 4-hydroxy group of the pyran ring establishes hydrogen-bonds with the two catalytic Asp^25^/Asp^25^′ at the cleavage site and the lactone moiety interacts directly with the flap region residues Ile^50^/Ile^50^′, replacing the structural water molecule found in the transition-state analogs crystallographic structures. Moreover, the groups appended to the 3-position occupy the S_1_ and S_2_ pockets, while the phenyl substituents on the S-phenyl moiety at C-3 filled the S_1_′ and S_2_′ pockets (Figure 15).

This second generation of protease inhibitors corresponds to those compounds most recently approved. They overcome some of the challenges exhibited by first-generation inhibitors including overcoming in part multidrug resistance strains. Atazanavir exhibits good oral bioavailability, permitting one dose per day. However, the long-term use of these compounds is associated with the induction of diverse metabolic syndromes such as dyslipidemia, insulin-resistance, and lipodystrophy/ lipoatrophy, as well as cardiovascular and cerebrovascular diseases [91].

The search for novel compounds after the approval of darunavir followed two different pathways. One the one hand, the search of new scaffolds embedding less peptide features and, on the other, modifications of existing compounds aimed at diminishing their secondary effects. Cyclic ureas like mozenavir belong to the first category, whereas derivatives of second-generation protease inhibitors with diverse functionalities like TMC-310911 belong to the second [92].

**Figure 16:**
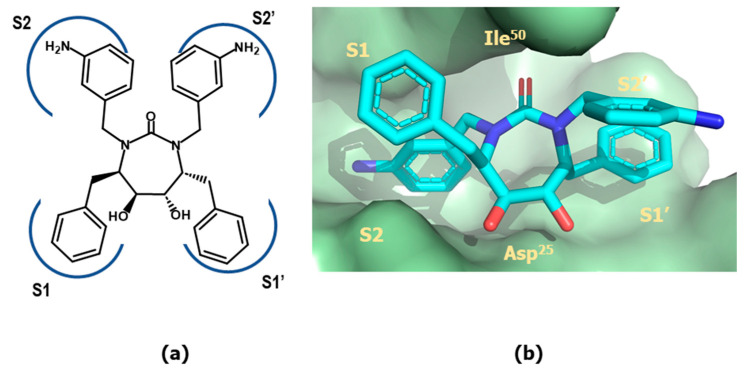
Binding mode of mozenavir (**57**) to the HIV protease. (**a**) schematic representation of ligand occupation of the diverse enzyme subsites. (**b**) 3D display of the ligand binding mode using the crystallographic structure (pdb entry 1DMP).

Cyclic ureas were designed after a key structural feature found in the 4-hydroxypyrones, i.e., the removal of a conserved water molecule mediating contacts between the carbonyl oxygens of the peptide inhibitors and the amide groups of Ile^50^/Ile^50^′ in the enzyme. Aimed at finding alternative scaffolds that occupy the binding site in a similar manner, extensive modeling studies were carried out. The results of these studies permitted to identify cyclic ureas as suitable scaffolds, capable to displace the structural water and to interact directly with Asp^25^/Asp^25^′, as well as to properly place their substituents into the different subsites of the binding cleft. One of the best compounds discovered is mozenavir (DMP-450) (**57**) (Figure 13). The X-ray crystallographic structure of mozenavir shows the diol-cyclic urea as a satisfactory scaffold capable to place the different substituents in the S_1_, S_2_, S_1_′ and S_2_′ pockets (see Figure 16) [93]. Unfortunately, the development of mozenavir was halted after early clinical trials due to a demonstrated poor pharmacokinetics profile. Cyclic ureas inspired other series of inhibitors. Close relatives of these compounds are the cyclic sulfonamides (**59**) or the closely related N-acyl azacyclic ureas (**58**) (Figure 13). This latter can be considered the result of the cyclization of linear aza backbone-modified isosteres previously used in first-generation HIV-1 inhibitors (**51**) [94]. Other cyclic scaffolds have also been tried including the 6-hydroxy-1,3-dioxin-4-one, 3-hydroxycyclohex-2-enone, tetronic acid, lactams, pyrrolidine or triazol [95]. Compounds of this series exhibit inhibitory activities in the nanomolar range, however their low oral bioavailability prevents their further development as therapeutic agents.

Darunavir and tipranavir have been used to develop novel inhibitors based on their high drug-resistance profiles. Interestingly, both have ability to inhibit protease dimerization necessary for protease activity. These compounds bind in such a way that the S_1_ and S_2_ sites are occupied with hydrophobic moieties, whereas S_1_′ and S_2_′ are occupied with moieties like, the 4-amino sulfonamide, designed to establish hydrogen bond interactions with backbone atoms of the enzyme. The candidate TMC-310911 (**60**) (Figure 13) was designed from darunavir by modification of the S_1_ moiety with a bulky hydrophobic moiety. The compound is orally available and exhibits nanomolar affinity for the HIV protease. Its development was discontinued after the second phase of clinical trials, as it failed to show greater benefits than its parent compound for the treatment of AIDS [96].

### 4.3. Thrombin Inhibitors

Blood clotting is one of the multiple interlinked steps of hemostasis, the process responsible to maintain the integrity of the circulatory system by keeping blood in a fluid state under physiologic conditions and arresting bleeding after vascular injury. Thrombin is a serine protease that plays a central role in the blood clotting process [97], performing diverse functions that help stabilize the formed clot and further seal up the wound. Specifically, thrombin participates in the conversion of soluble fibrinogen into fibrin as well as to the activation of upstream zymogens factors V, VIII, and XI, by specifically cleaving sequences Arg-Gly, which then further accelerate the clotting cascade by thrombin synthesis [97]. In addition, thrombin participates in the activation of factor XIII to stabilize the clot by favoring the formation of cross-linked bonds among the fibrin molecules. In the conversion of fibrinogen into fibrin, the N-terminus of both, the fibrinogen A and B chains is proteolytically cleaved to produce two short peptides of lengths 16 and 14 amino acids, labeled as fribrinopeptide A (FPA) and fibrinopeptide B (FPB), respectively. The resulting fibrin monomers polymerize end-to-end to form protofibrils, which in turn associate laterally to form fibrin fibers by lateral aggregation. Local deposition of fibrin forms an extensive meshwork that surrounds the aggregated platelets to form a stabilized clot that seals the site of vascular injury, preventing blood loss [98].

A deficient performance of the hemostatic system can be deleterious for a human being. Thrombosis or obstructive clotting is a pathological condition where blood clots are formed abnormally in a blood vessel. This occurs when a clot breaks free and travels around the body to finally obstruct a blood vessel, which unless treated quickly will lead to tissue necrosis in the area past the occlusion. Thrombosis is a common pathology underlying ischemic heart disease, ischemic stroke, and venous thromboembolism being major contributors to the global decease burden [99]. Accordingly, anticoagulants are a cornerstone of therapy for conditions associated with arterial and venous thrombosis and to reduce thromboembolic occurrence.

Due to the central role of thrombin in the coagulation process, it is considered a suitable target for developing therapeutic anticoagulants. The serpin small protein antithrombin is the native direct inhibition of thrombin that blocks its interaction with its substrates. Unfortunately, antithrombin cannot be used therapeutically due to its short half-life, together with the associated risks to properly control its concentration in blood, since there is no antidote to halt its function. Antithrombin acts slowly, but its activity can be significantly enhanced by heparin—a highly sulfated glycosaminoglycan—making it a useful modulator of its activity. Moreover, since the action of heparin can be reversed with protamine sulfate, it and its low molecular weight analogs have been the anticoagulant therapy of choice for many years. Despite its benefits, heparin may cause a severe immune reaction known as heparin-induced thrombocytopenia, leading to clot formation instead of preventing it.

The first direct thrombin inhibitor described for therapeutic use was hirudin, a 65-residue protein isolated from the salivary extracts of blood-sucking leeches, devoid of some of the shortcomings of antithrombin. Hirudin is the most potent natural inhibitor of thrombin described to date and works by specifically inhibiting the action of thrombin on fibrinogen. Moreover, hirudin action does not depend on antithrombin and does not trigger immune-mediated heparin-induced thrombocytopenia, making it a remedy to be used when heparin is contraindicated. However, despite its demonstrated performance of heparin as alternative therapy in specific cases, it lacks oral bioavailability and has a small therapeutic window that constitutes a major obstacle for chronic treatment of thromboembolic diseases [100]. Accordingly, efforts were put forward to design oral direct thrombin inhibitors. The process for their development is described below and represents an interesting example of peptidomimetic design [101].

The crystallographic structure of thrombin reveals that the enzyme is highly homologous to other serine proteases, with the characteristic catalytic triad constituted by residues Ser^195^, His^57^, and Asp^102^ necessary for the nucleophilic attack of the target peptide bond. A specific feature of thrombin that can be exploited to design specific inhibitors is the specificity of subsite S_1_ to accommodate arginine, with subsites S_2_ and S_3_ being more hydrophobic than in other serine proteases [102]. In addition, the protein presents two loops framing the active-site pocket that interact with both termini of the substrates to facilitate enzyme’s proteolytic function. Moreover, thrombin has two anion-binding exosites that are made up of surface exposed basic residues, used to interact specifically with negatively charged regions of thrombin cofactors and substrates. Hirudin is considered a bivalent inhibitor of thrombin because it binds to both the active site and to the anionic binding exosite [103]. Specifically, the N-terminus blocks the active-site of thrombin without occupying the S_1_ pocket, whereas the C-terminus binds to the fibrinogen exosite of the enzyme. Aimed at finding ligands that mimic hirudin epitopes as direct thrombin inhibitors, diverse peptides were designed. The design process was conceived in such a way that peptides preserved the bivalent nature of hirudin. These studies led to the discovery of the 20-residue peptide bivalirudin, a commercially available anticoagulant. Its sequence corresponds to the four first residues of hirudin N-terminus plus the twelve last residues of the C-terminus, joined by a spacer consisting in four glycines [104].

**Figure 17:**
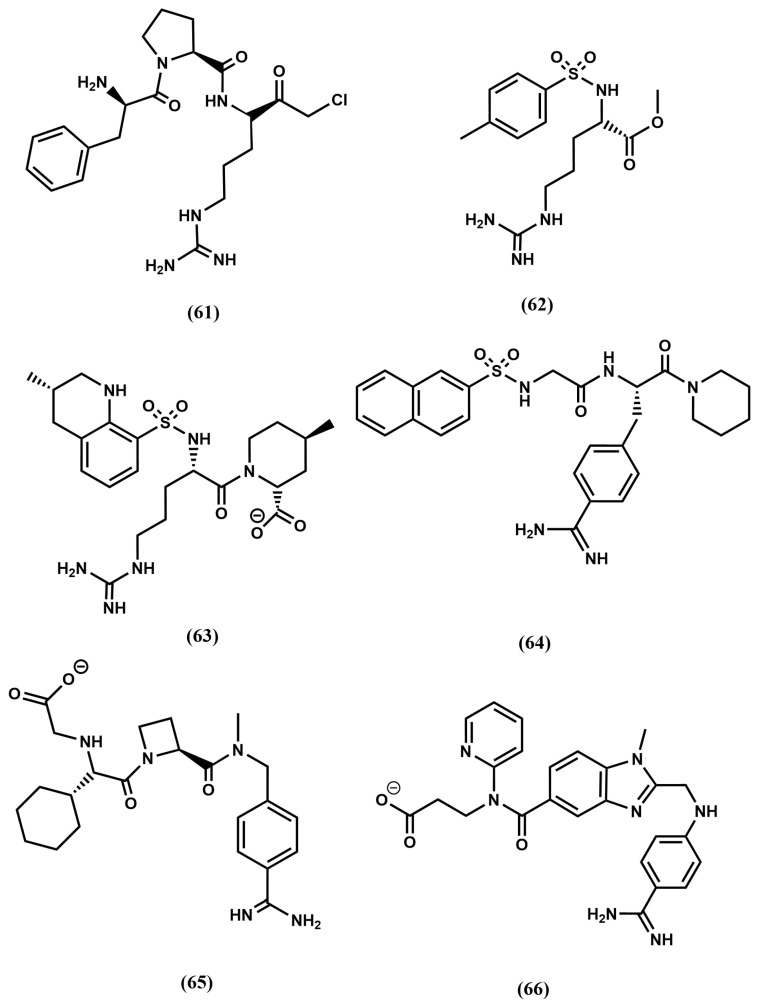
Chemical structures of diverse thrombin inhibitors (see text).

**Figure 18:**
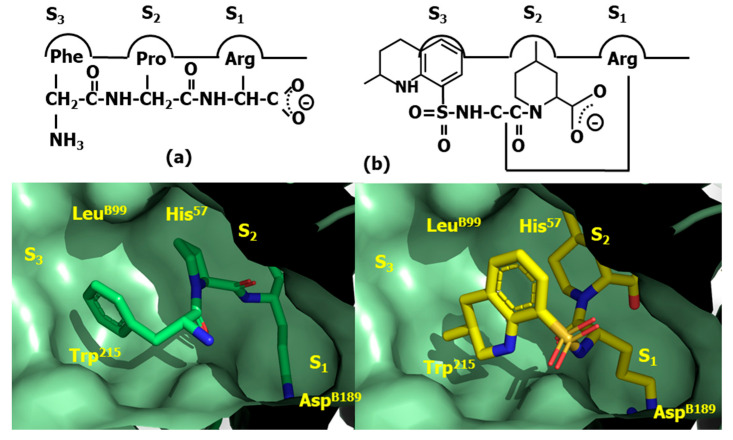
(**a**) Binding mode of PPACK (**61**) to thrombin: Schematic representation of ligand occupation of the diverse enzyme subsites and 3D display of the ligand binding mode using the crystallographic structure (pdb entry 1PPB). (**b**) Binding mode of argatroban (**63**) to thrombin: Schematic representation of ligand occupation of the diverse enzyme subsites and 3D display of the ligand binding mode using the crystallographic structure (pdb entry 4HFP).

Despite bivalirudin and its diverse constructs exhibit short half-life due to the scissile bond after the P_1_ arginine residue, which is slowly cleaved by thrombin, it represents a proof-of concept that thrombin can be directly inhibited by small molecules. A key discovery that facilitated the subsequent development of a small molecule, orally bioavailable direct thrombin inhibitors was the observation that the FPA fragment of antithrombin with sequence Arg-Val-Gly-Gly-Gly-Glu-Ala-Leu-Phe, exhibits antithrombotic properties. Analysis of the corresponding sequences across diverse species suggested that N- and C-terminal residues Arg and Phe, respectively, were essential to provide its inhibitory effect on thrombin. Synthesis and test of diverse peptide analogs led to the discovery of the tripeptide Phe-Val-Arg as the shortest analog preserving affinity for thrombin [105]. Despite the sequence does not correspond to any FPA fragment, the finding can be easily understood in terms of a β-turn conformation adopted by FPA when bound to thrombin, in such a way that residues at both termini are spatially close to each other when facing the binding site [106]. Subsequent structure–activity studies led to the discovery of the tripeptide DPhe-Pro-Arg with similar inhibitory potency [107]. Moreover, it was also found that the addition of electrophilic groups to these peptides produce irreversible inhibitors that mimic the transition state of the proteolytic cleavage of the peptide bond, increasing the affinity for the enzyme compared to their corresponding substrates [108]. Thus, substitution of the C-terminal ester DPhe-Pro-Arg by an aldehyde yielded the potent thrombin inhibitor efegatran. Similarly, the addition of a chloromethyl ketone moiety to the peptide yields the D-phenylalanyl-L-prolyl-L-arginyl chloromethylketone (PPACK) (**61**) [109] (Figure 17), a potent direct inhibitor of thrombin (Figure 18a). As can be seen, the arginyl side chain moiety plays the role of P_1_; the prolyl side chain plays of P_2_ and the D-phenylalanyl side chain plays of P_3_.

The quest for an oral small molecule peptidomimetics of DPhe-Pro-Arg was initiated in diverse laboratories following a medicinal chemistry approach. Thus, taking inspiration from the synthetic trypsin substrate tosylarginine methyl ester (TAME), (**62**) (Figure 17) [110] they reported the discovery of the high affinity non-covalent inhibitor argatroban (**63**) (Figure 17) [111]. The design process of this compound involved the modification of TAME by preserving the lysine side chain, P_1_; enlargement the hydrophobic moiety, P_3_ and the addition of a second hydrophobic moiety, P_2_ (see Figure 18b). The molecule is a potent inhibitor of thrombin, but cannot be administered orally due to its short half-life and its poor absorption in the gastrointestinal tract. In contrast, the compound presents the advantage of not being immunogenic compared to hirudin.

**Figure 19:**
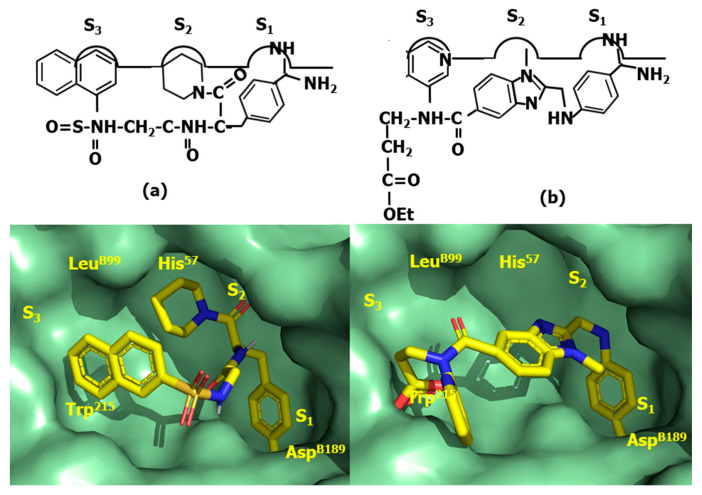
(**a**) Binding mode of NAPAP (**64**) to thrombin: Schematic representation of ligand occupation of the diverse enzyme subsites and 3D display of the ligand binding mode using the crystallographic structure (pdb entry 1ETS). (**b**) Binding mode of dabigatran (**66**) to thrombin: Schematic representation of ligand occupation of the diverse enzyme subsites and 3D display of the ligand binding mode using the crystallographic structure (pdb entry 8TQS).

Following a similar designing approach as for argatroban, led to the discovery of the low affinity direct thrombin inhibitor Nα-(2-naphthyl-sulphonyl-glycyl)-DL-p-amidinophenylalanyl-piperidine (NAPAP) (**64**) [112] (Figure 17). The compound is also influenced by TAME, featuring an enlarged hydrophobic moiety at P_3_ and the addition of a second hydrophobic moiety at P_2_, where the lysine moiety is modified, making use of the knowledge that benzamidine derivatives are inhibitors of thrombin was released in close succession [113] (Figure 19a).

Following a different path, megalatran (**65**) was designed as a direct peptidomimetic of the tripeptide DPhe-Pro-Arg [114]. Taking inspiration on previous structure–activity studies, the arginine side chain is replaced by a benzoimidine moiety; the proline by azetidine 2-carboxylic acid and the phenylalanine by cyclohexylglycine. Unfortunately, its oral bioavailability is low due to its poor absorption. Effort were put forward to improve it and the molecule was transformed into a double-prodrug through acetylation of the carboxylic acid group and the transformation of the basic amidino group into an amidoxime moiety to yield ximelagatran, that it is well absorbed in the small intestine and has an oral bioavailability of about 20% [115].

Finally, the third direct thrombin inhibitor commercially available, dabigatran (**66**) was designed from a careful analysis of the crystallographic structure of the complex NAPAP-thrombin [116]. The compound was discovered after a few cycles of chemical synthesis, X-ray crystallography and molecular modeling [117]. Dabigatram exhibits the benzoimidine moiety as P_1_; the N-methyl group as P_2_ and the pyridine as P_3_. Unfortunately, the compound exhibits low oral bioavailability (Figure 19b). Thus, following the same strategy as used with megalatran, the prodrug (dabigatran eterxilate) was developed through acetylation of the carboxylic acid group and amidation of the basic amidino group into a n-hexylamide, converting it thereby into a more lipophilic prodrug.

As can be seen, the development of orally active direct thrombin inhibitors was challenging due to the need to convert water-soluble, poorly absorbable, active site inhibitors into fat-soluble prodrugs that were then transformed back to the active drug after intestinal absorption.

### 4.4. Proteasome Inhibitors

Protein degradation is an essential function carried out by proteases under a strict regulation, since aberrant proteolysis underlies pathological conditions such as inflammation, cardiovascular diseases, cancer or neurodegeneration. The process may take place via two main mechanisms including the ubiquitin–proteasome system and the autophagy–lysosomal pathway [2]. The former takes place in the cytosol and primarily targets short-lived and misfolded proteins. In this process, proteins are labeled with ubiquitin molecules, marking them for breakdown by the proteasome, a large protein complex with multiple protease sites designed to process them into oligopeptides [118]. On the other hand, the autophagy–lysosomal pathway is responsible for degrading long-lived proteins, damaged organelles, and protein aggregates. In the process, these components are enclosed in a vesicle called an autophagosome, which fuses with a lysosome to break down its contents [119]. In recent years, inhibition of the ubiquitin–proteasome system has been exploited therapeutically for restricting cancer tumor growth. The rationale behind this assumption is that disturbance of protein degradation leads to an accumulation of poly-ubiquitinated proteins, which may result in the disruption of cellular processes, cell cycle arrest, the induction of apoptosis, leading to inhibition of tumor growth and angiogenesis. Moreover, evidence supports that cancer cells are more sensitive to proteasome inhibition than normal cells [120].

The proteasome is a multi-subunit enzymatic complex present in the nucleus and cytoplasm of eukaryotic cells composed of two regulatory units (19s) and a core unit (20s) that embeds several threonine protease active sites [121]. The 3D structure of the core particle reveals a large macromolecular complex with an overall cylindrical architecture that is defined by four coaxially stacked rings, forming a central pore. The outer two rings are made of α subunits (α1–α7) with multiple ubiquitin binding sites playing a regulatory role by recognition of polyubiquitinated proteins, facilitating their transfer to the catalytic sites. The inner two rings are made of β subunits (β1–β7) embedding the catalytic sites. The β1, β2, and β5 subunits contain the proteolytic active sites with caspase-like, tryptic-like, and chymotryptic-like specificities, respectively, conferring the proteasome the ability to cleave peptide bonds at the C-terminus of acidic, basic and hydrophobic amino acid residues, respectively. In addition to the constitutive proteasome, there are alternative forms, like the immunoproteasome or the thymoproteasome that are involved in routine proteolytic functions, antigen processing, and T-cell selection, presenting subtle differences in their structure [121].

**Figure 20:**
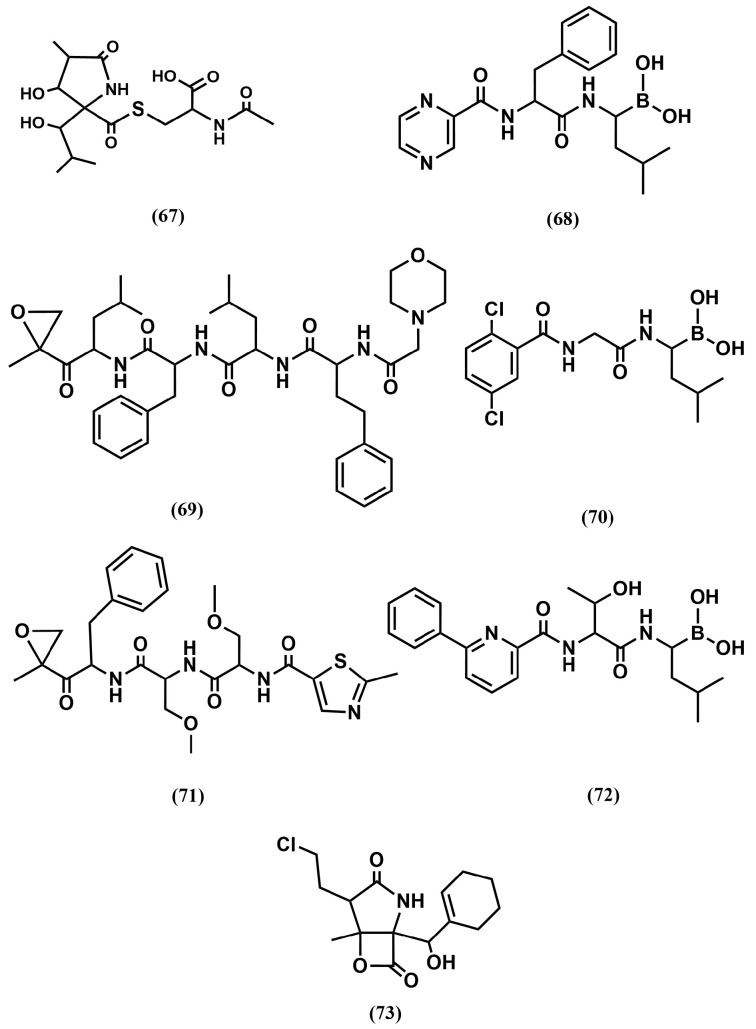
Chemical structures of diverse proteasome inhibitors (see text).

At the time of the discovery of the first proteasome inhibitors in the early 90s, the interest was more surrounding understanding the involvement of the proteasome in different cell functions than their possible use for therapeutic purposes. While several natural compounds—such as the microbial metabolite lactacystin (**67**) [122] (Figure 20), green tea polyphenols, and traditional medicinal triterpenes—have been identified as proteasome inhibitors, the first rationally designed inhibitor consisted of a peptide substrate bearing a warhead, i.e., an electrophilic functional group at its C-terminus, specifically engineered to form a covalent bond with the threonine side chain located in the catalytic sites of the proteasome. This strategy permitted the development of bortezomib (**68**), a peptide substrate with a boronic warhead that reversibly inhibits the chymotryptic-like site and also exhibits affinity for the caspase-like site (Figure 20). Most interestingly, it was found that the compound induces apoptosis in leukemic cell lines. This permitted the exploration of its use as therapeutic agent that led short after to its approval for the treatment of hematologic malignancies, namely multiple myeloma and mantle cell lymphoma [123].

Despite the success of the drug, its long-term use can lead to neuropathy of any grade. Moreover, a large number of patients fail to respond to this therapy, and almost all patients relapse from this drug. Mutations and/or over-expression of the protease subunits is a frequent cause of its resistance. Aimed at prolonging the duration of proteasome inhibition, a second-generation inhibitor carfilzomib (**69**), a peptide substrate with an epoxyketone warhead was developed a few years later and approved for the same therapeutic use [124] (Figure 20). It works as irreversible inhibitor and it is selective for the chymotryptic-like site, producing more sustained inhibition because synthesis of new proteasome complexes is required to reverse its action. Interestingly, the compound remains cytotoxic to some cells that are resistant to bortezomib. Moreover, carfilzomib exhibits lower rates of peripheral neuropathy than bortezomib, although it is cardiotoxic for some patients. The compound received accelerated approval in 2012 by the FDA as a single agent for the treatment of multiple myeloma.

Clinical responses to proteasome inhibitor therapy require frequent dosing and prolonged treatment and both, bortezomib and carfilzomib exhibit poor oral bioavailability and need to be administered intravenously. To overcome this drawback, ixazomib was subsequently developed (**70**) and approved in 2015 (Figure 20). It is a peptide substrate, reversible inhibitor with a boronic warhead with a similar pharmacological profile of bortezomib, but it exhibits improved pharmacokinetics, pharmacodynamics, and antitumor activity and exhibits good oral bioavailability [125].

Bortezomib, carfilzomib and ixazomib are the only proteasome inhibitors presently available in the marked for the treatment of multiple myeloma and mantle cell lymphoma. Despite a demonstrated effectiveness, their use in the clinic is challenging because they affect normal cells as well, leading to potential side effects such as immunosuppression and peripheral neuropathy. Moreover, cancer cells may develop resistance to proteasome inhibitors through various mechanisms, including mutations in the proteasome or upregulation of alternative protein degradation pathways [126]. These challenges stimulated the development of other inhibitors with improved properties. Thus, oprozomib (**71**), can be described as an orally bioavailable analog of carfilzomib. It is a tripeptide epoxyketone irreversible inhibitor, selective for the chymotryptic-like site of the proteasome [127] (Figure 20). The inhibitor, like its parent compound, does not exhibit peripheral neuropathy or cardiotoxicity, although it presents gastrointestinal toxicity. Unfortunately, it exhibits a shorter half-life time than its parent compound, fact that has delayed its advance in the clinic. Delanzomid (**72**) is an oral bioavailable peptidyl boronate. It is a reversible inhibitor of the proteasome with chymotrypsin-like activity that down-modulates NF-κB activity and the expression of several downstream effectors [128] (Figure 20). The compound was discontinued in phase I/II clinical trials due to overlapping toxicity profile with drugs like carfilzomib and ixazomib. Finally, marizomib (**73**) is the drug name of natural product salinosporamide A, isolated from a marine bacterium [129] (Figure 20). The compound is a β-lactone-γ-lactam, representing a different chemical class of compound in regard to the rest of proteasome inhibitors described above. It binds irreversibly, with capacity to inhibit the chymotryptic-like site, as well as the tryptic-like and caspase-like sites. It is not cross-resistant with bortezomib, with a short half-life and a large distribution volume. Its poor oral bioavailability makes that requires to be administered intravenously. Interestingly, it crosses the blood–brain barrier, unlike the rest of proteasome inhibitors and it was then considered for the treatment of glioblastoma. Unfortunately, the results of a clinical study do not reflect any benefit [130].

## 5. Discussion and Conclusions

Throughout this report, we emphasized the involvement of proteases in diverse physiological processes. On the one hand, protein degradation is key function of proteases, relevant to food digestion and intracellular protein turnover, but proteases act as signaling molecules regulating a variety of physiological processes, through the exercise of their proteolytic activity on diverse substrates. We also highlighted the use of protease inhibitors as therapeutic agents, mimicking nature regulatory mechanisms. A simple way to design protease inhibitors is to add a warhead to a substrate to produce an irreversible inhibitor, as in the case of bortezomib [123]. However, the challenge of using irreversible inhibitors regards their poor selectivity, likely ablating off-target proteases. In addition, due to the poor pharmacological profiles of peptides, the preferred drugs to seek are reversible small molecule inhibitors [6]. To help this design and considering that protease substrates largely bind in extended conformations, isostere substitutions at the scissile bond, mimicking the transition state geometry can easily produce a first generation of inhibitors [17]. These compounds normally require subsequent modifications to produce a second-generation of compounds with their pharmacokinetic profiles improved. To illustrate the rationale of this methodology, the present report includes a short section describing well-developed medicinal chemistry strategies to modify peptide substrates into peptidomimetics [131,132]. The development of protease inhibitors is illustrated in the present report by means of four successful case studies, selected among many others, with special focus on their discovery process and improvement. Three of them concern a physiological process regulation, whereas the fourth one regards protein degradation. As described below, today these compounds represent important classes of drugs with large market sizes and with high steady compound annual growth rate (CAGR) expectations.

The first case study regards the development of ACE inhibitors. These compounds are available in the market since the early 80s for the treatment of hypertension and congestive heart failure. They are also prescribed today for the treatment of diabetes-related conditions, such as diabetic nephropathy, stroke prevention, and proteinuria in chronic kidney disease. After more than 40 years from their commercialization, ACE inhibitors represent the second largest therapeutic class in the antihypertensive market, just behind the calcium channel blockers with a global size market of US$ 7.1 billion in 2024 [133]. Rising rates of cardiovascular disease incidence, increased aging population, clinical effectiveness and safety profile, prevalence of diabetes, regulatory endorsement and guidelines, makes the ACE inhibitors market size to grow steadily in recent years. Specifically, it is estimated the market size to reach a global value of US$ 9.6 billion by 2029 [133] at a CAGR of 5.3%.

The second case study regards the design of HIV protease inhibitors as antivirals [134]. Compounds like ritonavir, atazananvir or tipranavir approved by the turn of the century, transformed the HIV infection from a life-threatening illness to a manageable chronic disease [78]. According to the World Health Organization (WHO) an estimated 40.8 million individuals were living with an HIV infection at the end of 2024, with the WHO Africa region accounting for two thirds of them [76]. The success of the introduction of retrovirals in the market in the mid-90s was miraculous, with a reduction in the number of AIDS-related deaths by two thirds from 1994 [76]. The impact on the community was so crucial that it moved the Time magazine to honor Dr. David Ho, a pioneer of retroviral therapy, as Man of the Year for 1996. The global HIV drugs market size was US$ 36.11 billion in 2024 [135], but with the challenges due to an increasing prevalence of AIDS disease, especially in Africa, it is anticipated to drive market growth. Accordingly, it is expected the global HIV drugs market to reach US$ 58.24 billion by 2032, at a CAGR of 6.2% [135].

The third case study regards thrombin inhibitors. These are drugs used as anticoagulant agents, prescribed to prevent the formation of a blood clot or thrombosis. Venous thromboembolism, the third common cardiovascular disease after acute coronary syndrome and stroke. In addition, thrombosis can be initiated at the sites of an atherosclerotic lesion in arteries leading to acute myocardial infarction and stroke. As explained above, traditional anticoagulants include heparin, low molecular weight heparins and warfarin, covering about 50% of the market. At present, better treatment alternative anticoagulant drugs include direct thrombin inhibitors, factor Xa inhibitors, factor IX inhibitors, tissue factor inhibitors and novel vitamin K antagonists, being the two first the drugs most widely used of this group [136]. These oral available drugs are extensively used for stroke prophylaxis in patients with non-valvular atrial fibrillation; for treatment of venous thromboembolism; and also, for the secondary prophylaxis of acute coronary syndromes [137]. The global anticoagulants market size was estimated in US$ 35.9 billion in 2024, being around 5% the direct thrombin inhibitors share. In the current global blood thinners market scenario, there are a number of factors providing major impetus to the growth of these drugs [138]. On the one hand, there is a rising prevalence of cardiovascular diseases, such as Deep Vein Thrombosis, Pulmonary Embolism, and Atrial Fibrillation and, on the other, the increased awareness with respect to cardiovascular health and the possible repercussions or negative impact together with the appearance of novel oral anticoagulants. Accordingly, the global market is expected to grow at a CAGR of 4.8%, reaching US$ 47.1 billion by 2030 [138].

Finally, the fourth case study concerns proteasome inhibitors for the treatment of cancer. This is a class of cytotoxic medications that demonstrate anticancer capability by inhibiting the chymotrypsin-like enzymatic activity of the proteasome, which is involved in cellular breakdown of proteins. The current applications of proteasome inhibitors are in oncology, for the treatment of mantle cell lymphoma and multiple myeloma [123], but there are diverse clinical trials underway to expand its clinical applications. The Global Proteasome Inhibitors Market size was valued in US$ 10.8 billion in 2024 and expected to grow for diverse reasons [139]. First, global cancer incidence is rising due to aging populations and lifestyle factors; the use of these inhibitors for the treatment of other diseases is under investigation; increasingly targeting emerging markets for expansion. Accordingly, the Global Proteasome Inhibitors Market is projected to grow at a CAGR of 7.1% during the forecast period of 2025 to 2032 [139].

In addition to the case studies described in the present report, there are many other successful stories of small molecule protease inhibitors design [8]. However, not all the targets are successful first hand, with specific challenges needed to be taken into consideration. One key aspect is to have a full understanding of the biological roles of the target and understand the degree of cross reactivity that inhibitors may exhibit to closely related members of an enzyme family. The story behind the design of matrix metalloprotease (MMP) inhibitors exemplifies this point. Members of this subclass of proteases were considered as interesting targets to combat cancer, due to their role in extracellular matrix degradation. Accordingly, several inhibitors were developed in the mid-90s, but failed in advanced clinical trials because severe off-target effects, due to the lack of selectivity and no apparent clinical benefit, due to overlapping mechanisms of diverse MMPs in matrix turnover [140]. This led to the development of MPPs to be largely abandoned. Today, we know that MMPs initiate multifactorial proteolytic cascades, creating new substrates, activating or suppressing other MMPs, and generating signaling molecules in a process known as network proteolysis [141]. Despite the complexity, many of us consider the design of MMPs as an opportunity to develop novel medicines and there several programs working in this direction [142,143].

In summary, we underlined the relevance of developing protease inhibitors as therapeutic drugs, describe their design as an exemplifying model of peptidomimetics, highlighting the use of peptide bond isostere replacement as a thriving methodology to discover novel compounds. We also acknowledged the need of a deeper understanding of the target biology before any attempt to design protease inhibitors, exemplified by the early efforts to design MMPs inhibitors. Finally, we showcased selected examples of successful design through four diverse case studies and showed the relevance of these compounds in the drug market, even years after their commercialization.
